# Spatiotemporally Regulated Pro-/Anti-Inflammatory Transition via Reactive Oxygen Species-Sensitive Core–Shell Hydrogel for Diabetic Infected Wound Healing

**DOI:** 10.34133/bmr.0432

**Published:** 2026-10-15

**Authors:** Jian Zhang, Yan Lin, Yan Liu, Wentao Wang, Yuanyuan Li, Ben Zhong Tang, Xin Wang

**Affiliations:** ^1^ Department of Plastic Reconstructive Surgery & Hand Microsurgery, Ningbo No.6 Hospital, Ningbo, Zhejiang 315040, China.; ^2^ Ningbo Clinical Research Center for Orthopedics, Sports Medicine & Rehabilitation, Ningbo, Zhejiang 315040, China.; ^3^ College of Science, Nanjing Forestry University, Nanjing, Jiangsu 210037, China.; ^4^ State Key Laboratory for Diagnosis and Treatment of Severe Zoonotic Infectious Diseases, Key Laboratory for Zoonosis Research of the Ministry of Education, Institute of Zoonosis, and College of Veterinary Medicine, Jilin University, Changchun, Jilin 130062, China.; ^5^ Guangdong Basic Research Center of Excellence for Aggregate Science, School of Science and Engineering, The Chinese University of Hong Kong (Shenzhen), Longgang, Shenzhen, Guangdong 518172, China.

## Abstract

Diabetic infected wounds remain difficult to heal because bacterial colonization, biofilm formation, excessive reactive oxygen species (ROS), and persistent inflammation mutually reinforce one another in the diabetic wound microenvironment. In diabetic wounds, the temporal immune switch is often impaired, resulting in oxidative stress accumulation and poor vascularized tissue repair. Therefore, an ideal wound dressing should not merely kill bacteria or suppress inflammation independently, but should coordinate antibacterial intervention and immune remodeling according to the evolving stages of wound healing. Here, we constructed a ROS-responsive core–shell hydrogel, BBT-Py^+^ NPs@SchB@CSH, to achieve spatiotemporally ordered therapy for diabetic infected wounds. The outer shell was loaded with BBT-Py^+^ nanoparticles, an aggregation-induced emission luminogen (AIEgen) with near-infrared imaging capability and synergistic photothermal–photodynamic antibacterial activity against *Staphylococcus aureus* and biofilms. The inner core encapsulated schisandrin B (Sch B), a natural antioxidant and immunomodulator that scavenges excessive ROS and promotes macrophage polarization from the pro-inflammatory M1 phenotype toward the reparative M2 phenotype. The ROS-sensitive interface enabled the infected microenvironment and laser-triggered ROS to accelerate outer-shell degradation, allowing early antibacterial action to be followed by Sch B-mediated immunoregulatory repair. In vitro and in vivo evaluations demonstrated that BBT-Py^+^ NPs@SchB@CSH effectively disrupted bacterial biofilms, reduced bacterial burden, alleviated oxidative inflammatory stress, promoted collagen deposition and angiogenesis, and accelerated diabetic infected wound closure. This study presents an “outer-layer antibacterial, inner-core immunomodulatory repair” strategy that matches therapeutic function to the temporal requirements of infected diabetic wound healing.

## Introduction

Chronic diabetic wound healing is a highly coordinated biological process in which inflammation plays a critical double-edged role [1,2]. In the early stage of infection, when pathogens such as *Staphylococcus aureus* invade, a moderate local inflammatory response serves as the first line of defense by recruiting neutrophils and macrophages to eliminate pathogens and restrain bacterial growth [3–7]. However, in the diabetic state, this finely tuned response is often disrupted. Hyperglycemia-induced microcirculatory dysfunction and immune impairment can prolong inflammatory activation beyond its antimicrobial function, leading to persistent inflammation and delayed healing [8–11]. Most current strategies for chronic diabetic wounds mainly aim to suppress excessive inflammation while underestimating the importance of a controlled early inflammatory phase for bacterial clearance and wound initiation [12]. Therefore, a key challenge in treating diabetic infected wounds is how to preserve the early anti-infection immune response while timely resolving excessive inflammation, thereby enabling a coordinated transition from pro-inflammatory to anti-inflammatory signaling over time. This motivates a staged therapeutic design in which the early inflammatory phase is harnessed for bacterial clearance, followed by timely inflammation resolution to prevent the wound from progressing into a chronic nonhealing state.

To achieve this “antibacterial first, then anti-inflammatory” regulation in a controlled manner, there is an urgent need for a drug delivery system that can work synergistically to facilitate this functional switch [13,14]. Recently, hydrogel dressings with timed release capabilities have attracted attention due to their unique structural advantages. Unlike homogeneous hydrogels, which struggle to perform both antibacterial and anti-inflammatory roles simultaneously, core–shell structures allow different functional modules to be loaded into separate layers, providing an ideal platform for sequential spatiotemporal treatment [15–17]. The outer shell quickly releases antimicrobial agents to control early infection, while the core stores anti-inflammatory drugs that are released once the outer shell degrades [18–20]. This design, based on compartmentalized drug loading and staged release, not only prevents interference between components but also enables precise spatiotemporal control of the transition from antibacterial to anti-inflammatory effects [21]. Importantly, adding a reactive oxygen species (ROS)-sensitive interface as a linker allows high ROS levels in the infection microenvironment to trigger outer-layer degradation, facilitating the timely release of inner-layer drugs and providing a promising solution for meeting the phased needs of diabetic-infected wound healing [22,23]. Accordingly, this double-layer design is introduced to preserve the early host-defense response, enable bactericidal intervention during the inflammatory phase, and then switch to anti-inflammatory repair once the infection burden is reduced.

Long-term use of antibiotics can lead to the development of antibiotic resistance in pathogenic bacteria. Therefore, reducing or even avoiding the use of antibiotics has become a crucial approach to managing diabetic-infected wounds. Photoreactive antibacterial therapies have gained marked attention due to their noninvasive nature, spatiotemporal controllability, and low potential for inducing drug resistance [24]. These therapies employ photosensitizers to generate ROS [photodynamic therapy (PDT)] or localized hyperthermia [photothermal therapy (PTT)] upon exposure to specific-wavelength light, thereby effectively eliminating antibiotic-resistant bacteria and biofilms [25]. However, traditional photosensitizers (such as porphyrins and indocyanine green) often suffer from aggregation-caused quenching (ACQ) in aqueous or high-concentration environments, thereby reducing their photodynamic efficacy and limiting their applications [26]. Photosensitizers with aggregation-induced emission (AIE) characteristics overcome this limitation: Their twisted molecular conformations enhance fluorescence in the aggregated state and maintain efficient ROS generation [27–29]. This enables AIE photosensitizers to exert strong photodynamic/photothermal effects even when embedded in a 3-dimensional (3D) hydrogel network, effectively disrupting bacterial membranes and degrading biofilm extracellular polymeric substances under laser irradiation, making them ideal tools for combating antibiotic-resistant infections [30].

Implementing appropriate anti-inflammatory interventions during the wound healing phase is a critical step in the treatment of diabetic-infected wounds [31,32]. Traditional Chinese medicine, with its multi-target and multi-pathway regulatory characteristics, offers unique advantages for modulating the complex inflammatory microenvironment [33]. Schisandrin B (Sch B), an active component of Schisandra chinensis, has been shown to inhibit pro-inflammatory cytokine release and induce macrophage polarization toward the anti-inflammatory M2 phenotype, thereby promoting the secretion of pro-repair factors [34]. Furthermore, its polyphenolic hydroxyl groups enable direct free-radical scavenging, effectively neutralizing residual ROS and alleviating oxidative stress damage [35,36]. Compared to single-target chemical anti-inflammatory drugs, the “anti-inflammatory–antioxidant–immunomodulatory” synergy of Sch B is more consistent with the complex pathological microenvironment of diabetic-infected wounds [37,38].

In this study, we developed an ROS-responsive, zwitterionic core–shell hydrogel system (BBT-Py^+^ NPs@SchB@CSH) designed for precise, staged treatment of diabetic bacterial-infected wounds (Fig. 1). The outer shell contains BBT-Py^+^ nanoparticles (NPs), providing synergistic photothermal–photodynamic antibacterial activity to efficiently eliminate bacteria and biofilms in the early stages of infection. The inner core, containing the natural antioxidant Sch B, provides ROS-scavenging and immunomodulatory functions to alleviate oxidative stress and drive macrophage polarization from the M1 to the M2 phenotype in the late repair stage. The layers are connected by ROS-sensitive bonds, and the high ROS levels in the infected microenvironment trigger outer-layer degradation, enabling the sequential release of the inner-layer drug. Through systematic in vitro and in vivo evaluations, we investigated the hydrogel’s antibacterial performance, ROS-scavenging capacity, and immunomodulatory mechanisms, providing a novel and promising strategy with translational potential for the clinical management of diabetic-infected wounds. In other words, the treatment follows a clear spatiotemporal order: In the early inflammatory phase, BBT-Py^+^ NP-mediated photothermal/photodynamic antibacterial action eliminates pathogens and biofilms; then, after bacterial burden is reduced, Sch B is released to clear excessive inflammation and prevent the wound from becoming chronic.

**Fig. 1.**
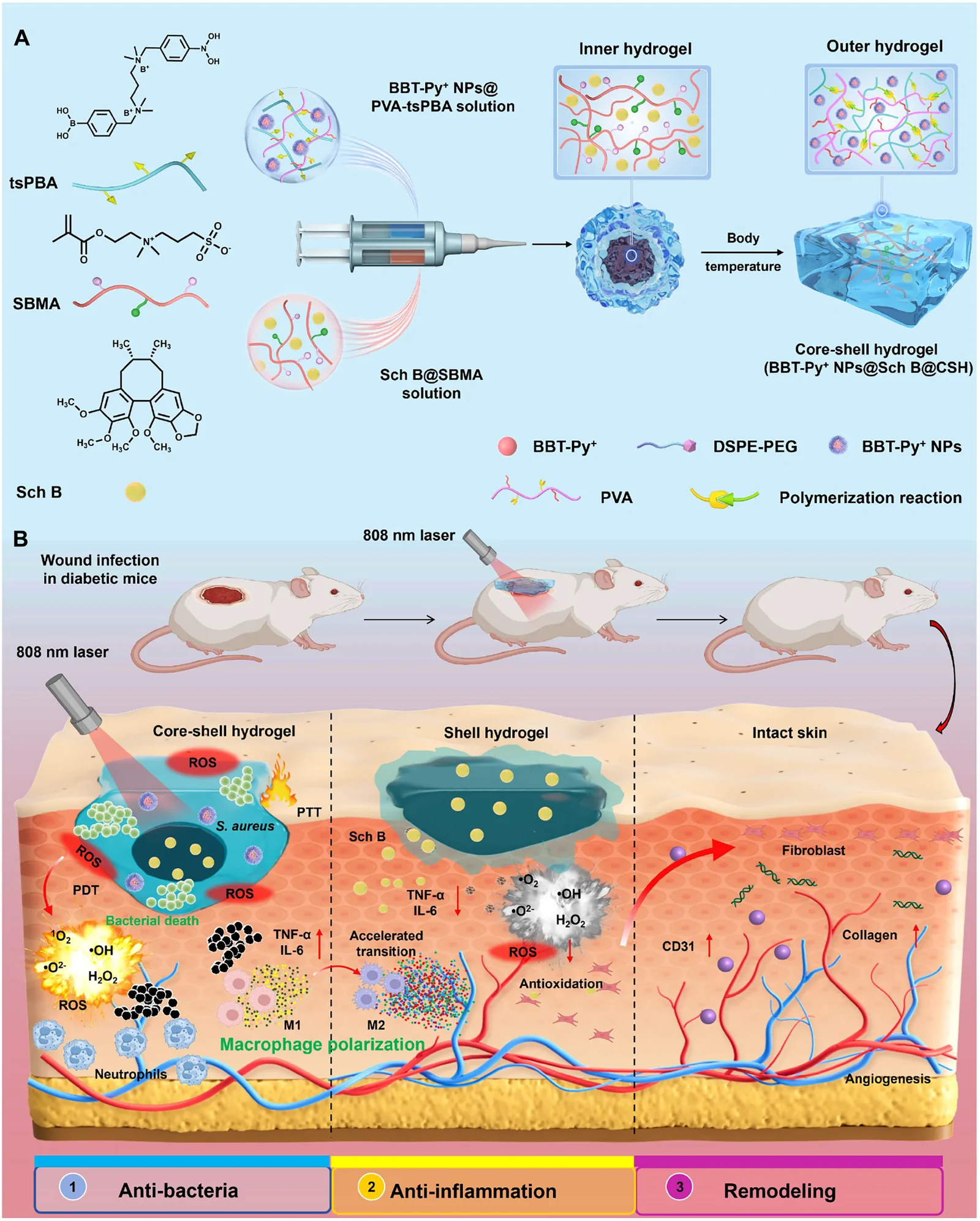
(A) Schematic diagram of the preparation of the core–shell gel. (B) Application of the core–shell hydrogel for the treatment of diabetic-infected wounds.

## Materials and Methods

### Materials

4-(Bromomethyl)phenylboronic acid (containing varying amounts of anhydride), *N*,*N*,*N*′,*N*′-tetramethyl-1,3-propanediamine, and [2-(methacryloyloxy)ethyl]dimethyl-(3-sulfopropyl) (SPE) were purchased from Aladdin Biochemical Technology Company (Shanghai, China). Sch B was purchased from TargetMol. 2′,7′-Dichlorodihydrofluorescein-diacetate (DCFH-DA) was purchased from Beyotime Biotechnology (Shanghai, China). Dimethyl sulfoxide [DMSO; analytical reagent (AR), ≥99.5%] and tetrahydrofuran (THF; AR, ≥99.5%) were purchased from China National Pharmaceutical Group Chemical Reagent Co. Ltd.

### Preparation of BBT-Py^+^ NPs

BBT-Py^+^ (1 mg) and DSPE-PEG_2000_-NH_2_ (amino-polyethylene glycol-phospholipid) (2 mg) were dissolved in 1 ml of THF and then dispersed by sonication. The solution was slowly injected into 9 ml of deionized water under stirring (600 rpm) and further sonicated for 7 min. The mixture was freeze-dried to remove THF and then redissolved in deionized water to obtain a dispersion of BBT-Py^+^ NPs.

### Construction of core–shell hydrogel

To prepare TSPBA [N^1^-(4-boronobenzyl)-N^3^-(4-boronophenyl)-N^1^, N^1^, N^3^, N^1^-tetramethylpropane-1, 3-diaminium], 0.2 g of *N*,*N*,*N*′,*N*′-tetramethyl-1,3-propanediamine (1.5 mM) and 1 g of 4-(bromomethyl)phenylboronic acid (4.6 mM) were dissolved in 40 ml of dimethylformamide (DMF). The mixture was stirred at 60 °C for 24 h. The reaction solution was then poured into 200 ml of THF to precipitate the product. The crude product was washed with THF 3 times and subsequently vacuum-dried to obtain pure TSPBA. Paolyvinyl alcohol (PVA)-TSPBA was prepared via dynamic phenylboronic acid-transcyclohexanediol cross-linking: 0.5 ml of PVA (1%, w/v) and 0.5 ml of TSPBA (2.5%, w/v) were mixed and gelled at room temperature within 30 s.

To prepare the inner gel, 2.6 g of SBMA [3-[dimethyl-[2-(2-methylprop-2-enoyloxy)ethyl]azaniumyl]propane-1-sulfonate] was dissolved in 5 ml of deionized water at room temperature under stirring until complete dissolution. Then, a Sch B stock solution was added to achieve a final concentration of 50 μg/ml, and the mixture was stirred for 30 min to obtain the Sch B@SBMA composite solution. The core–shell hydrogel was constructed using a layered injection method: First, 50 μl of Sch B@SBMA was injected into the center of the mold (diameter 8 mm) and allowed to gel for 5 to 10 min; subsequently, 50 μl of BBT-Py^+^ NPs@PVA-TSPBA was slowly injected around the core and allowed to cross-link at room temperature for 15 min to form a complete core–shell structure.

### Characterization

The hydrodynamic particle size and zeta potential of BBT-Py^+^ NPs were measured using a laser particle size analyzer (Malvern Zetasizer Nano ZS-90) with an equilibration time of 120 s. The hydrogel was fractured in liquid nitrogen, freeze-dried for 24 h, and sputter-coated with gold. The cross-sectional morphology was observed using scanning electron microscopy (SEM; Hitachi S-4800) at an accelerating voltage of 5 kV. The specific characterization methods can be found in the Supplementary Materials.

### Statistical analysis

All data were presented as mean ± standard deviation (SD). Statistical analyses were carried out using GraphPad Prism 9.0 (GraphPad Software) and Origin 2024 (OriginLab). Comparisons between 2 groups were analyzed using the unpaired Student’s *t* test. In contrast, comparisons among multiple groups were performed using either 1-way or 2-way analysis of variance (ANOVA), followed by Bonferroni’s post hoc test for multiple comparisons. Differences were considered statistically significant at *P < 0.05, **P < 0.01, and ***P < 0.001.

## Results and Discussion

### Design of the BBT-Py^+^ NPs@SchB@CSH

The synthesis of BBT-Py^+^ was based on the article published by our group [39]. The detailed synthesis method of the aggregation-induced emission luminogens (AIEgens) (BBT-Py^+^) is illustrated in Figs. S1 and S2, which exhibit near-infrared-II (NIR) fluorescence imaging, as well as photothermal and photodynamic effects. This study successfully constructed a multifunctional nano-hydrogel system, BBT-Py^+^ NPs@SchB@CSH. Using DSPE-PEG_2000_ as a mediator, BBT-Py^+^ could self-assemble into uniformly shaped NPs (Fig. 2A). Transmission electron microscopy (TEM) and dynamic light scattering (DLS) confirmed an average NP size of approximately 193 nm (Fig. 2B and C) with a polydispersity index (PDI) of 0.22, indicating uniform distribution of NPs and a zeta potential of +8.29 mV (Fig. S3A). Stability tests showed that there were no significant changes in hydrodynamic particle size within 7 d (Fig. S3B). These NPs exhibited significant changes in NIR-II fluorescence intensity in environments with varying water content (Fig. 2D) and remained stably dispersed within the hydrogel matrix. Digital photographs confirmed the successful formation of a stable core–shell hydrogel with a visually distinguishable compartmentalized structure (Fig. 2E). SEM revealed that the NP-loaded hydrogel retained a well-defined porous network (Fig. 2F), which may provide a favorable microenvironment for cell infiltration and nutrient exchange.

**Fig. 2.**
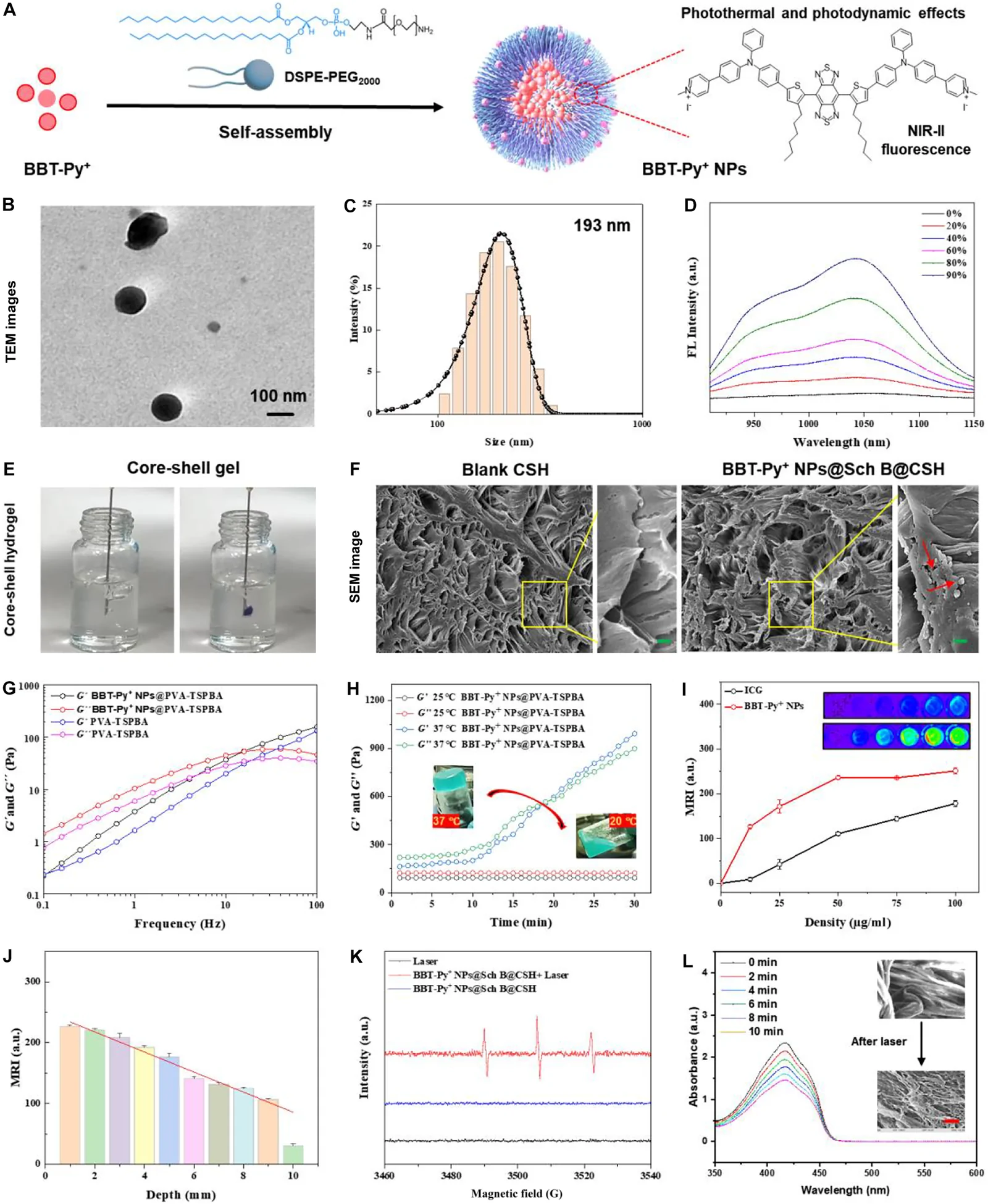
(A) Schematic illustration of BBT-Py^+^ self-assembly into BBT-Py^+^ NPs. (B) Representative TEM image of BBT-Py^+^ NPs. Scale bar, 100 nm. (C) DLS size distribution of BBT-Py^+^ NPs. (D) Fluorescence emission spectra of BBT-Py^+^ NPs in the NIR-II region (950 to 1,150 nm) at different water fractions. (E) Photographs showing the visual appearance of the precursor solutions and the final BBT-Py^+^ NPs@SchB@CSH hydrogel. (F) Representative SEM images of CSH (left) and BBT-Py^+^ NPs@SchB@CSH (right). Scale bar, 1 μm. (G and H) Storage modulus (*G*′) and loss modulus (*G*″) of BBT-Py^+^ NPs@PVA-TSPBA, BBT-Py^+^ NPs@PVA-TSPBA, and PVA-TSPBA hydrogels as a function of frequency (0.1 to 100 Hz) (temperature: 37 °C). (I) Fluorescence imaging signal intensity of BBT-Py^+^ NPs at different concentrations (0 to 100 μg/ml). Inset: Corresponding fluorescence images. (J) Fluorescence signal intensity of BBT-Py^+^ NPs@SchB@CSH at different depths. (K) EPR spectra of BBT-Py^+^ NPs@SchB@CSH before and after laser irradiation, confirming ROS generation. (L) Fluorescence absorption spectra of BBT-Py^+^ NPs@SchB@CSH at different time points after laser irradiation. Inset: SEM images before and after laser treatment.

Rheological frequency sweep measurements revealed that the storage modulus (*G*′) of both BBT-Py^+^ NPs@PVA-TSPBA composite hydrogel and pristine PVA-TSPBA hydrogel was lower than the loss modulus (*G*″) in the low-frequency region, demonstrating a viscous fluid-dominated behavior. As the oscillation frequency increased, *G*′ rose continuously and surpassed *G*″, indicating that elastic performance became the dominant mechanical property of the hydrogel (Fig. 2G). Time-sweep rheological tests of BBT-Py^+^ NPs@PVA-TSPBA composite hydrogel under different temperatures were further conducted. At 25 °C, the hydrogel exhibited nearly constant low moduli with *G*′ always smaller than *G*′, verifying that the material existed as a free-flowing sol at room temperature. When incubated at 37 °C (simulating physiological temperature), both *G*′ and *G*″ increased sharply after 10 min, and *G*′ exceeded *G*″ at approximately 20 min, which corresponded to a prominent sol-to-gel phase transition (Fig. 2H). The pristine PVA-TSPBA hydrogel displayed identical temperature-dependent gelation profiles in time-sweep characterization (Fig. S4). Collectively, these results confirm that the PVA-TSPBA matrix possesses outstanding thermoresponsive gelation capacity originating from dynamic boronate ester bonds, endowing the hydrogel with core merits of injectability at room temperature and in situ gelation under physiological temperature.

Fluorescence imaging evaluation revealed that BBT-Py^+^ NPs demonstrated superior imaging capability compared to the clinically used contrast agent indocyanine green (ICG), with an imaging penetration depth of up to 10 mm (Fig. 2I and J). This offers reliable technical support for the precise imaging and localization of deep bacterial-infected wounds. Electron paramagnetic resonance (EPR) spectroscopy confirmed that, upon 808-nm laser irradiation, the multifunctional nano-hydrogel efficiently generated singlet oxygen (^1^O_2_) (Fig. 2K). Concurrently, the hydrogel’s absorption spectrum and microscopic morphology were significantly altered post-irradiation (Fig. 2L), suggesting that the photothermal effect can induce dynamic changes in the gel network, enabling controlled and enhanced drug release. Furthermore, the absorbance of the BBT-Py^+^ NPs@SchB@CSH irradiation group at 410 nm gradually decreased with prolonged laser exposure. Quantitative analysis (Fig. S5) indicated that loading Sch B into the hydrogel system did not significantly compromise its photodynamic performance.

### Evaluation of antioxidant and photothermal properties

Endowing biomedical materials with efficient ROS-scavenging capacity is clinically significant for alleviating oxidative damage, regulating inflammation, and promoting wound healing. We systematically evaluated the performance of the inner-layer gel (Sch B@SBMA) as a multifunctional nanozyme in modulating oxidative stress. To elucidate its potential to alleviate oxidative stress at the wound site, the total antioxidant capacity of Sch B@SBMA was quantitatively assessed (Fig. 3A). The results demonstrated a clear Sch B concentration-dependent enhancement in antioxidant activity, suggesting that its efficacy can be precisely tuned by modulating the Sch B loading. We further investigated the scavenging efficiency of Sch B@SBMA against H_2_O_2_ and 2 representative reactive radicals (·OH and ·O_2_^−^) (Fig. 3B to D). At a Sch B concentration of 50 μg/ml, the material exhibited optimal scavenging activity against all 3 types of ROS, confirming its broad-spectrum and efficient ability to scavenge ROS.

**Fig. 3.**
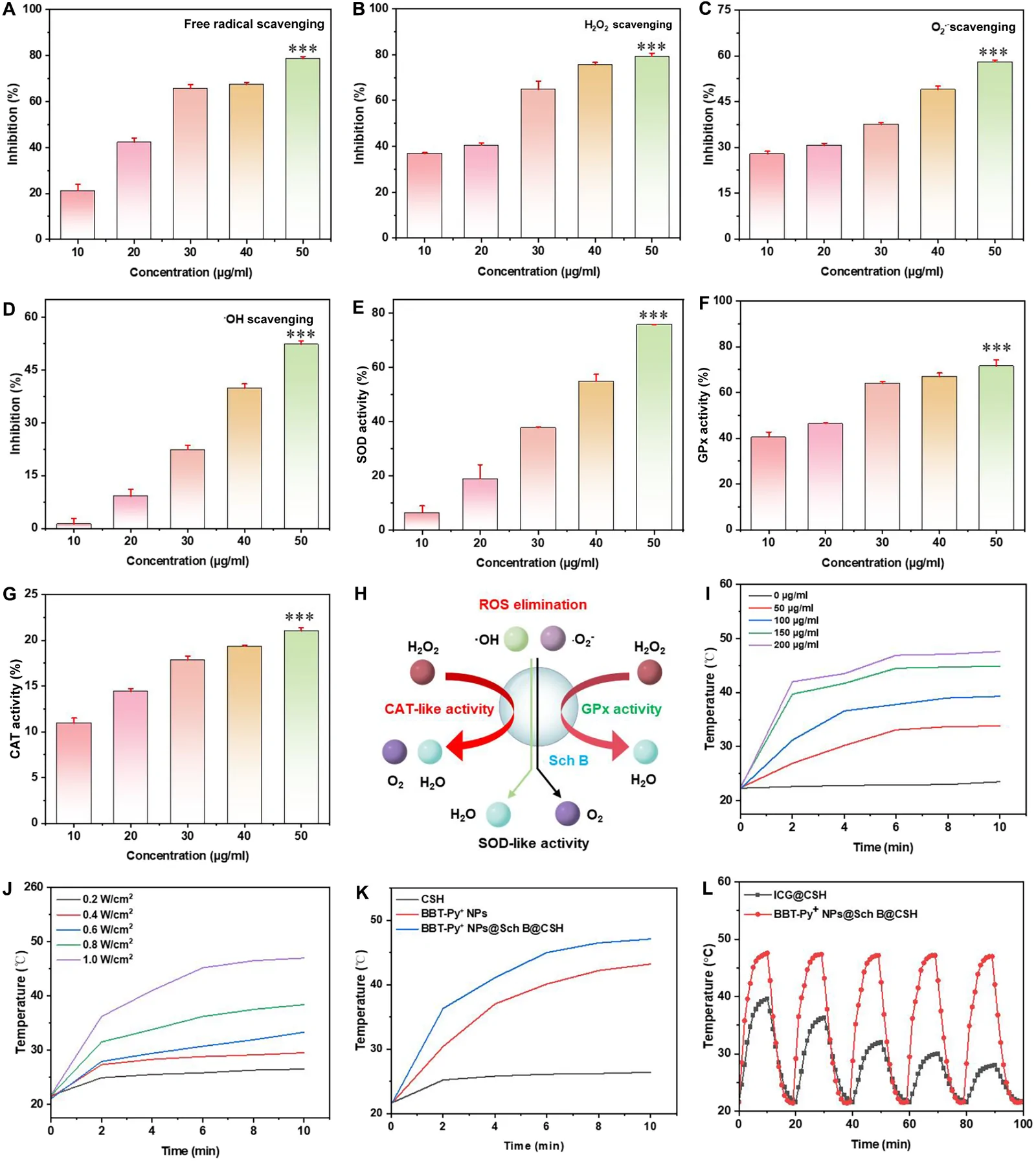
(A) Total antioxidant capacity, (B) H_2_O_2_ scavenging ability, (C) ·O_2_^−^ scavenging ability, (D) ·OH scavenging ability, (E) SOD activity measurement, (F) GPx activity measurement, and (G) CAT activity measurement of Sch B@SBMA containing different concentrations of Sch B. (H) Schematic representation of the antioxidant mechanism of Sch B. (I) Photothermal heating curves of different concentrations of BBT-Py^+^ NPs under 808-nm laser irradiation. (J) Temperature elevation curves of BBT-Py^+^ NPs under different laser power densities. (K) Temperature elevation curves of CSH, BBT-Py^+^ NPs, and BBT-Py^+^ NPs@SchB@CSH. (L) Heating and cooling curves of BBT-Py^+^ NPs@SchB@CSH and ICG solution under 808-nm laser irradiation. (I to L) Irradiation was performed at 808-nm wavelength, 1.0 W cm^−2^ power density, and for 10 min. Error bars represent mean ± SD. ***P < 0.001.

To systematically evaluate the photothermal properties and conversion efficiency of BBT-Py^+^ NPs, experiments were conducted using an 808-nm laser as the excitation source. Infrared thermal imaging and temperature change profiles demonstrated that the photothermal conversion efficiency of the BBT-Py^+^ NP aqueous solution was concentration-dependent (Fig. 3I and Fig. S6A). Deionized water, used as a negative control, exhibited no significant temperature fluctuation under identical irradiation conditions. In contrast, at a laser intensity of 1.0 W cm^−2^, the temperature of a 200 μg/ml BBT-Py^+^ NP solution rapidly increased to approximately 47.8 °C, indicating excellent photothermal conversion ability (Fig. 3J and Fig. S6B). To investigate the influence of laser irradiation on different materials, CSH, BBT-Py^+^ NPs, and BBT-Py^+^ NPs@SchB@CSH were continuously irradiated with an 808-nm laser for 10 min, and their photothermal responses were compared. Among the 3, the BBT-Py^+^ NPs@SchB@CSH composite hydrogel exhibited the most pronounced photothermal response (Fig. 3K and Fig. S7). Photostability is a key indicator for evaluating the potential of materials in PTT applications. Therefore, we further investigated the photothermal stability of BBT-Py^+^ NPs@SchB@CSH. The composite hydrogel was subjected to 5 consecutive “on/off” cycles under 808-nm laser irradiation (1.0 W cm^−2^), and temperature changes were monitored in real time. The temperature profile showed no significant decrease after 5 cycles, indicating excellent photothermal stability suitable for long-term applications (Fig. 3L and Fig. S8).

To further elucidate the enzymatic antioxidant mechanism of Sch B@SBMA, we evaluated its ability to mimic 3 key endogenous antioxidant enzymes. Concentration-dependent experiments showed that Sch B@SBMA significantly mimicked the activities of superoxide dismutase (SOD), glutathione peroxidase (GPx), and catalase (CAT) (Fig. 3E to G). To explore its CAT-like catalytic mechanism, we focused on the effect of Sch B concentration on this activity. Steady-state kinetic analysis, performed by varying the H_2_O_2_ concentration, revealed that the catalytic reaction followed typical Michaelis–Menten kinetics. With H_2_O_2_ as the substrate, the Michaelis constant (*K*_m_) was 76.87 mM, and the maximum reaction rate (*V*_max_) was 2.38 × 10^−4^ Ms^−1^ (Fig. S9). In conclusion, the Sch B component within the hydrogel system plays a pivotal role in eliminating excess free radicals and alleviating oxidative stress damage in the wound microenvironment (Fig. 3H).

### Immunomodulatory properties of BBT-Py^+^ NPs@SchB@CSH

Because the drugs can be adsorbed on the hydrogel surface and retain their anti-inflammatory activity, direct quantitative determination of Sch B release kinetics under the illumination sequence will yield deviations. Therefore, we utilized the antioxidant function experiment after laser action to indirectly confirm this temporal sequential mechanism. Consistent with the in vitro antioxidant assays, the BBT-Py^+^ NPs@SchB@CSH composite hydrogel following laser irradiation exhibited significantly enhanced ROS-scavenging ability compared to the nonirradiated group (Fig. S10A to D). Given the detrimental effects of excessive ROS on physiological systems, we first conducted a systematic in vitro biosafety evaluation of the 3 materials: Sch B@SBMA, BBT-Py^+^ NPs@PVA-TSPBA, and BBT-Py^+^ NPs@SchB@CSH. Cell counting kit-8 (CCK-8) cytotoxicity assays showed that, under different treatment conditions, the viability of RAW 264.7 macrophages remained above 90% (Fig. S11A), indicating no obvious cytotoxicity. Live/dead staining further corroborated these findings, confirming the good biocompatibility of all 3 materials (Fig. S11B). To explore the in vitro cytoprotective effect of BBT-Py^+^ NPs@SchB@CSH, we used the ROS-specific fluorescent probe DCFH-DA in an oxidative stress model induced by lipoteichoic acid (LPS). Fluorescence imaging revealed that, compared to the positive control group, cells treated with BBT-Py^+^ NPs@SchB@CSH + Laser exhibited significantly reduced green fluorescence intensity, indicating excellent ROS-scavenging ability under laser irradiation and effective protection of macrophages from oxidative damage (Fig. 4A and Fig. S12). This potent ROS-scavenging performance under laser irradiation provides a foundation for regulating the inflammatory response and accelerating wound healing at the infection site. Quantitative flow cytometry further confirmed that the ROS-scavenging ability of the BBT-Py^+^ NPs@SchB@CSH + Laser group was significantly superior to that of the nonirradiated BBT-Py^+^ NPs@SchB@CSH group (Fig. 4B and C), confirming its effectiveness in alleviating intracellular oxidative stress and potentially protecting cells from ROS-mediated damage.

**Fig. 4.**
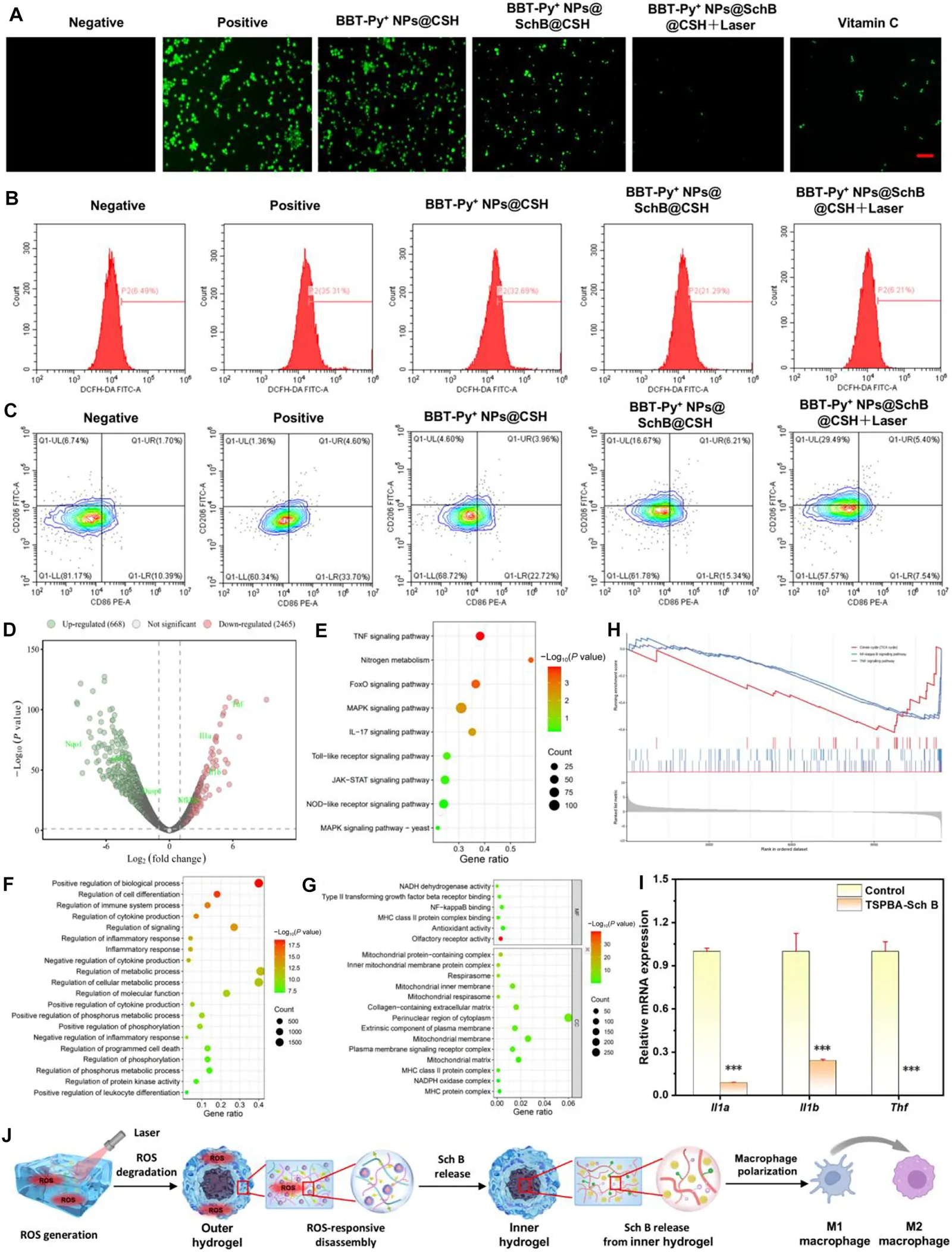
(A) Fluorescence images showing intracellular ROS levels in different treatment groups (negative control, positive control, BBT-Py^+^ NPs@CSH, BBT-Py^+^ NPs@SchB@CSH, BBT-Py^+^ NPs@SchB@CSH + Laser, Vitamin C). (B) Flow cytometric detection of intracellular ROS levels in different treatment groups using DCFH-DA staining. (C) Flow cytometric analysis of the proportions of M1 (CD86^+^) and M2 (CD206^+^) subtypes of macrophages in different treatment groups. (D) Volcano plot of DEGs in different treatment groups, showing up-regulated (green), down-regulated (red), and no significant change (gray) genes. (E) Bubble plot of KEGG pathway enrichment analysis of DEGs. (F) Bubble plot of GO functional enrichment analysis of DEGs in biological processes. (G) GO enrichment analysis of DEGs in molecular functions and cellular components. (H) GSEA results for the top 3 enriched signaling pathways. (I) qRT-PCR analysis of relative mRNA expression levels of pro-inflammatory factors (*II1a*, *II1b*, and *Tnf*). (J) Schematic illustration of the ROS-responsive drug release and macrophage polarization regulation mechanism of BBT-Py^+^ NPs@SchB@CSH under laser irradiation: Laser-induced ROS generation and outer hydrogel degradation trigger inner hydrogel disintegration and Sch B release, promoting macrophage polarization from M1 to M2 phenotype for anti-inflammatory effects. Error bars represent mean ± SD. ***P < 0.001.

To elucidate the molecular mechanism by which BBT-Py^+^ NPs@SchB@CSH regulates the inflammatory response, we performed transcriptome sequencing analysis. Compared to the control group, 3,133 differentially expressed genes (DEGs) were identified in the BBT-Py^+^ NPs@SchB@CSH + Laser treatment group, with 668 significantly up-regulated and 2,465 significantly down-regulated (Fig. 4D). Kyoto Encyclopedia of Genes and Genomes (KEGG) pathway enrichment analysis indicated that these DEGs were significantly enriched in various inflammation-related pathways, such as the tumor necrosis factor (TNF), mitogen-activated protein kinase (MAPK), interleukin-17 (IL-17), Toll-like receptor, Janus kinase–signal transducer and activator of transcription (JAK–STAT), and nucleotide-binding oligomerization domain (NOD)-like receptor signaling pathways (Fig. 4E). Gene Ontology (GO) functional enrichment analysis further revealed that these DEGs were primarily involved in the positive regulation of biological processes, including cell differentiation, immune system processes, cytokine production, and signal transduction. Notably, multiple DEGs were significantly enriched in negative regulation of inflammatory response, negative regulation of cytokine production, and phagocytosis-related processes, suggesting that this composite hydrogel exerts anti-inflammatory effects by modulating these pathways (Fig. 4F). Molecular function enrichment analysis showed that the DEGs were mainly associated with antioxidant activity, the major histocompatibility complex class II (MHC II) protein complex, and mitochondrial electron transfer-related functions (Fig. 4G). Gene set enrichment analysis (GSEA) further confirmed that inflammation-related signaling pathways were significantly down-regulated in the BBT-Py^+^ NPs@SchB@CSH + Laser treatment group (Fig. 4H).

Quantitative real-time polymerase chain reaction (qRT-PCR) validated the transcriptome results, showing that mRNA expression levels of pro-inflammatory factors *Il1a*, *Il1b*, and *Tnf* were significantly decreased in the TSPBA-Sch B treatment group (Fig. 4I). In summary, BBT-Py^+^ NPs@SchB@CSH likely exerts its anti-inflammatory and pro-healing effects through a synergistic “ROS-responsive release and immunomodulation” mechanism: Under 808-nm laser irradiation, the outer hydrogel generates ROS and degrades, triggering the disintegration of the inner hydrogel and the release of Sch B. The released Sch B then efficiently scavenges excess ROS in the wound microenvironment, alleviating oxidative stress damage. Concurrently, it modulates multiple inflammation-related signaling pathways to promote macrophage polarization toward the anti-inflammatory M2 phenotype, thereby inhibiting the expression of pro-inflammatory factors and ultimately achieving the dual biological effects of anti-inflammation and wound healing (Fig. 4J). This synergistic mechanism presents a promising approach for developing novel smart nanohydrogels for the targeted treatment of infected wounds.

### Evaluation of antibacterial efficacy

To systematically evaluate the efficacy of the BBT-Py^+^ NPs@SchB@CSH composite hydrogel against bacterial biofilms, we conducted experiments using an *S. aureus* biofilm model. Comparing the antibacterial effects of the control group, high-power (HP) laser irradiation group, BBT-Py^+^ NPs@SchB@CSH, BBT-Py^+^ NPs@SchB@CSH + low-power (LP) laser irradiation, and BBT-Py^+^ NPs@SchB@CSH + HP (Fig. 5A and B), we found that HP laser irradiation alone showed no significant antibacterial activity. The BBT-Py^+^ NPs@SchB@CSH alone treatment group exhibited a clear inhibitory effect. In addition, combining HP irradiation with the composite hydrogel demonstrated the strongest antibacterial efficacy, suggesting a synergistic effect between PTT and the composite hydrogel. SEM observations confirmed this conclusion. Compared to the intact and smooth bacterial and biofilm structures in the control group, bacteria in the BBT-Py^+^ NPs@SchB@CSH + HP treatment group showed significant lysis, severe biofilm structural damage, and cytoplasmic leakage (Fig. 5C), directly reflecting the destructive effect of this combined treatment. To further quantify the bactericidal effect, we performed live/dead bacterial staining. The BBT-Py^+^ NPs@SchB@CSH + HP treatment group exhibited the strongest red fluorescence signal (Fig. 5D), indicating the highest bacterial mortality and further confirming the significant synergistic antibacterial effect. DCFH-DA fluorescence probe detection showed that both the BBT-Py^+^ NPs@SchB@CSH + LP and + HP groups presented strong ROS fluorescence signals (Fig. 5E), confirming that oxidative stress plays a key regulatory role in the antibacterial process. Further SEM analysis revealed that, after BBT-Py^+^ NPs@SchB@CSH + HP treatment, bacterial adhesion density was significantly reduced, and some bacteria exhibited abnormal morphology (Fig. 5F), suggesting that this composite hydrogel combined with LP laser irradiation may inhibit bacterial adhesion, disrupt cell membrane integrity, and thereby exert a biofilm-inhibitory effect.

**Fig. 5.**
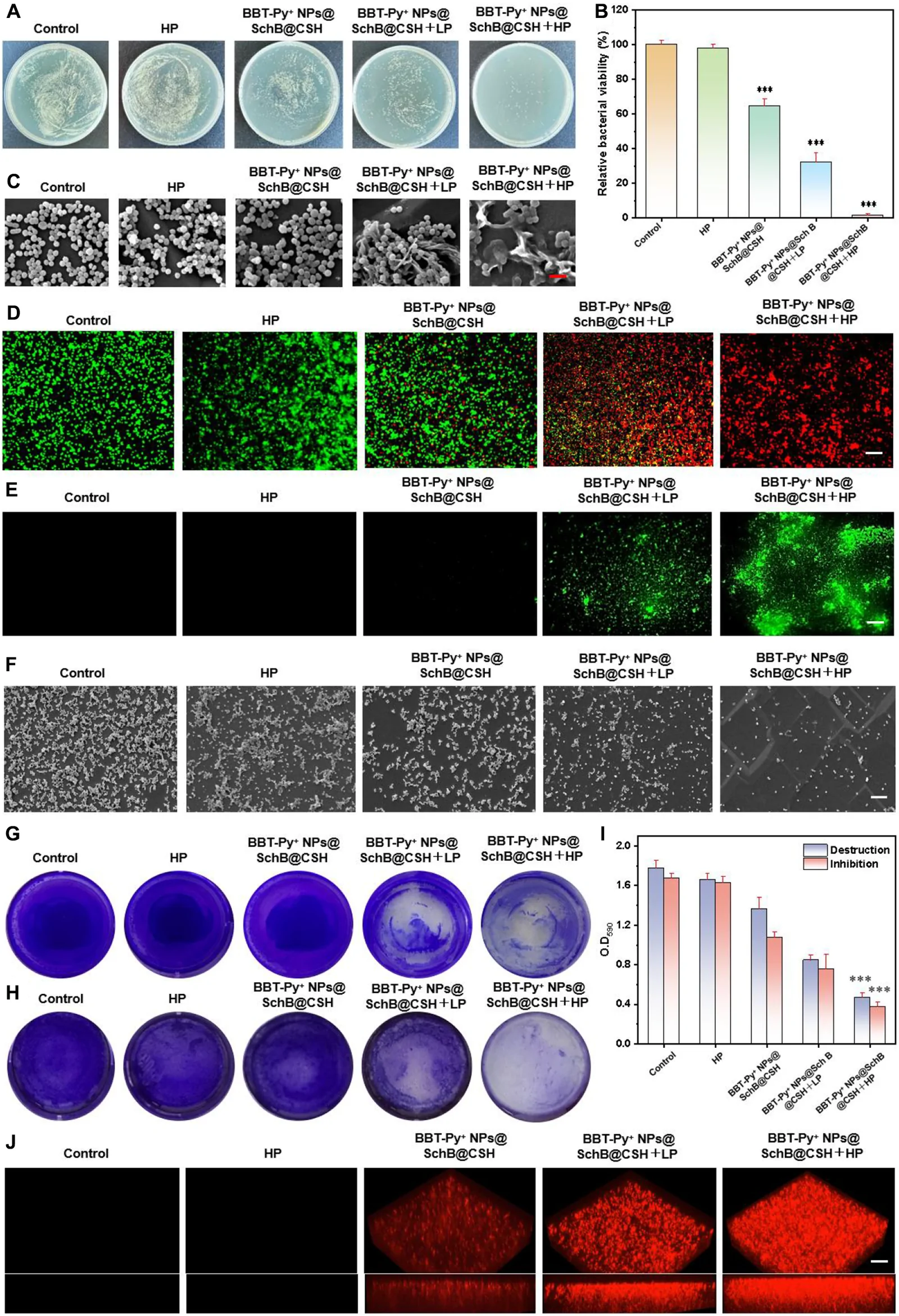
(A) Photographs of agar plates treated with blank LB, HP, BBT-Py^+^ NPs@SchB@CSH, BBT-Py^+^ NPs@SchB@CSH + LP, and BBT-Py^+^ NPs@SchB@CSH + HP, and (B) quantitative analysis of *S. aureus*. (C) Representative SEM images of *S. aureus* after different treatments. Scale bar, 2 μm. (D) Representative fluorescence images of *S. aureus* stained with SYTO 9 and PI. Scale bar, 100 μm. (E) Detection of bacterial oxidative stress using the ROS probe DCFH-DA after different treatments. Scale bar, 100 μm. (F) SEM images of *S. aureus* adhesion after 7 d of different treatments. (G) Crystal violet staining images of mature biofilms after corresponding treatments. (H) Crystal violet staining images of mature biofilms after different treatments. (I) Absorbance of residual crystal violet after different treatments. (J) Dead staining of *S. aureus* biofilms under different treatment interventions. (A, C to H, and J) Irradiation was performed at 808-nm wavelength, 1.0 W cm^−2^ power density, and for 10 min. Scale bar, 100 μm. ***P < 0.001.

Microbial biofilms possess complex 3D network structures, extracellular matrices, and intercellular signaling systems. These features collectively promote bacterial community growth, colonization, survival, and the development of antibiotic resistance, contributing to the difficulty in eradicating bacterial infections. Therefore, we further verified the antibiofilm capabilities of the control, HP, BBT-Py^+^ NPs@SchB@CSH, BBT-Py^+^ NPs@SchB@CSH + LP, and BBT-Py^+^ NPs@SchB@CSH + HP groups. Crystal violet staining was used to evaluate the effect of each group on bacterial biofilm dispersion. The results indicated that the BBT-Py^+^ NPs@SchB@CSH + HP group had a significant inhibitory and destructive effect on bacterial biofilms (Fig. 5G to I and Fig. S13). Propidium iodide (PI) staining was used further to evaluate antibacterial activity against bacteria within the biofilm. The control and HP groups showed no obvious bright red fluorescence, indicating intact biofilm structures and high bacterial viability. In contrast, the BBT-Py^+^ NPs@SchB@CSH + LP group and the BBT-Py^+^ NPs@SchB@CSH + HP group showed obvious red fluorescence, with the latter exhibiting stronger intensity (Fig. 5J), suggesting that the photothermal effect induced by BBT-Py^+^ NPs@SchB@CSH can effectively eliminate bacteria within the biofilm. Bacterial dry weight measurements showed that the biomass of the untreated control biofilm was 12.9 mg. In comparison, the dry weight of biofilms treated with the BBT-Py^+^ NPs@SchB@CSH + LP group and the BBT-Py^+^ NPs@SchB@CSH + HP group was reduced to 9.3 and 5.6 mg, respectively, further confirming that the combination of BBT-Py^+^ NPs@SchB@CSH and laser irradiation significantly reduces biofilm biomass, with HP irradiation being more effective (Fig. S14).

### Study on antibacterial mechanisms

To deeply elucidate the molecular mechanism by which BBT-Py^+^ NPs@SchB@CSH promotes bacterial biofilm dispersion, we conducted transcriptome sequencing analysis. Compared to the control group, the BBT-Py^+^ NPs@SchB@CSH + Laser treatment group identified 1,205 DEGs, with 1,084 significantly down-regulated and 121 significantly up-regulated (Fig. 6A). KEGG pathway enrichment analysis indicated that these DEGs were mainly enriched in signaling pathways closely related to amino acid and energy metabolism, such as the ATP-binding cassette (ABC) transporter pathway, arginine biosynthesis, alanine/aspartate/glutamate metabolism, valine/leucine/isoleucine biosynthesis, nitrogen metabolism, and sulfur metabolism (Fig. 6B). GO functional enrichment analysis further revealed that these DEGs were primarily involved in key biological functions such as protein metabolic processes, carbohydrate metabolic processes, amide metabolic processes, peptide biosynthetic processes, and organic acid metabolic processes (Fig. 6C). Metabolic pathways such as oxidative phosphorylation, the tricarboxylic acid (TCA) cycle, and aminoacyl-tRNA biosynthesis were also significantly enriched (Fig. 6D), suggesting that BBT-Py^+^ NPs@SchB@CSH may mediate bacterial biofilm dispersion by regulating bacterial metabolic processes.

**Fig. 6.**
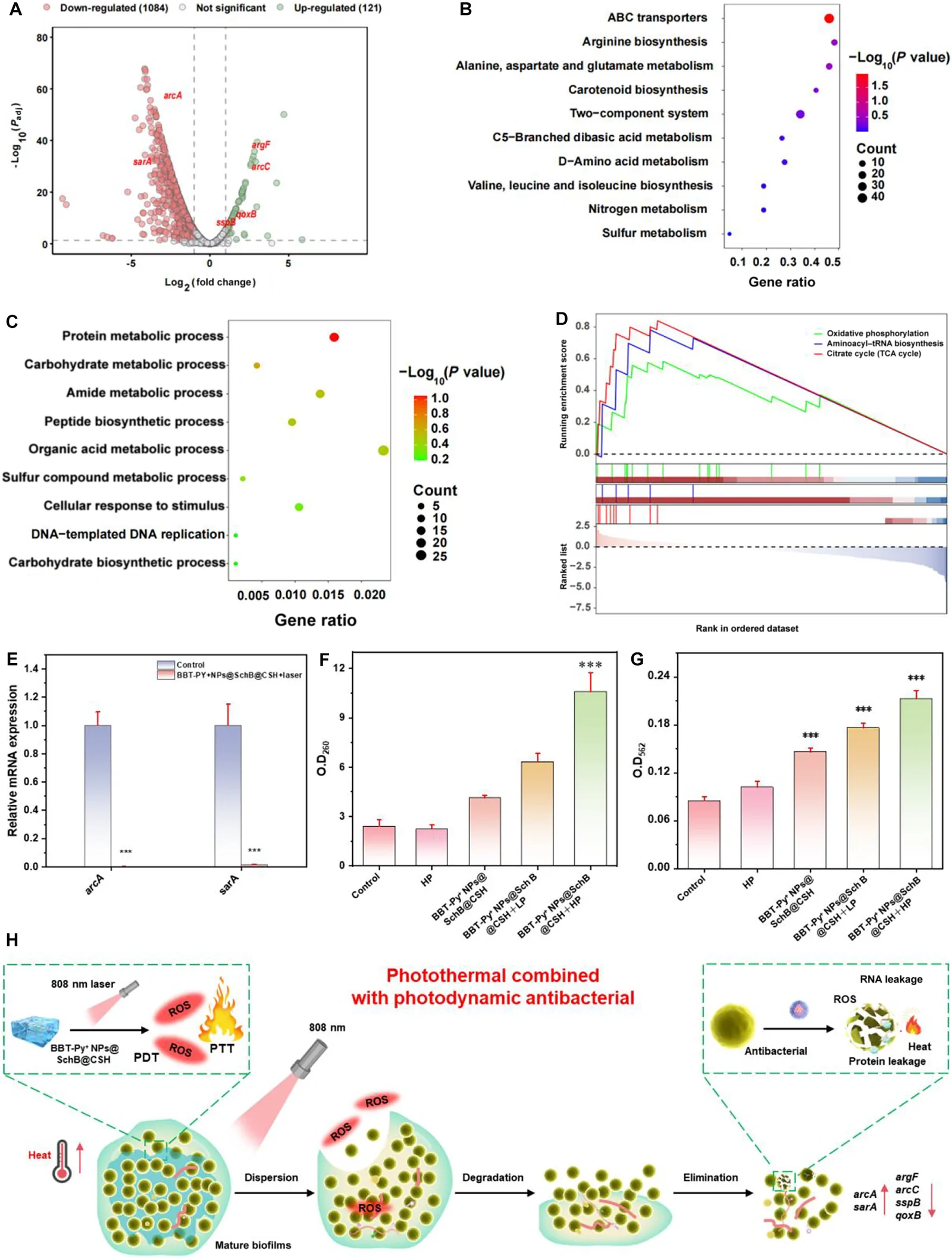
(A) Volcano plot of DEGs between the BBT-Py^+^ NPs@SchB@CSH + HP and untreated groups. (B) KEGG enrichment analysis of DEGs. (C) GO pathway enrichment analysis of DEGs. (D) GSEA results for the top 3 enriched signaling pathways. (E) qPCR analysis of *arcA* and *sarA* gene expression. Effects of different treatments on the extracellular nucleic acids and total proteins of *S. aureus* (F) and bacterial membranes (G). (H) Schematic diagram of the proposed antibacterial mechanism involving membrane disruption. Error bars represent mean ± SD. ***P < 0.001.

To further validate this mechanism, we performed targeted qRT-PCR. Notably, compared to the untreated control group, the expression levels of energy metabolism-related genes (*arcA* and *sarA*) in the BBT-Py^+^ NPs@SchB@CSH treatment group were significantly down-regulated (Fig. 6E). This suggests that the composite hydrogel may indirectly mediate bacterial biofilm dissociation by inhibiting the expression of genes involved in energy metabolism. Concurrently, key genes involved in arginine biosynthesis and degradation (*argF* and *arcC*) were significantly up-regulated (Fig. S15A and B), suggesting a potential disruption of bacterial amino acid metabolic balance, which may inhibit biofilm formation and promote dispersion. These results indicate that BBT-Py^+^ NPs@SchB@CSH can effectively regulate the transcription of genes related to amino acid synthesis and metabolism, thereby modulating bacterial biofilm dispersion. The bacterial cell membrane, as a key barrier maintaining structural integrity and normal physiological functions, directly determines bacterial survival.

To explore the effect of BBT-Py^+^ NPs@SchB@CSH on the bacterial cell membrane, we conducted nucleic acid and bicinchoninic acid assay (BCA) protein leakage assays. The results showed that, compared to other treatment groups, leakage of bacterial nucleic acids and proteins was most significant in the BBT-Py^+^ NPs@SchB@CSH + HP treatment group (Fig. 6F and G), indicating that the composite hydrogel combined with HP laser irradiation severely damages bacterial cell membrane integrity, leading to leakage of intracellular contents and ultimately inducing bacterial death. In conclusion, the BBT-Py^+^ NPs@SchB@CSH composite hydrogel system achieves potent antibacterial effects through the synergistic action of photothermal and photodynamic effects. It generates ROS and thermal effects that disrupt the 3D structure of mature bacterial biofilms and damage bacterial cell membrane integrity, causing leakage of intracellular components, and effectively removes excess ROS at the wound site, further degrading the biofilm matrix. This multi-pathway synergistic effect (Fig. 6H) provides an experimental and mechanistic basis for its application in the treatment of infected wounds.

### Evaluation of intra-body diabetic infection-induced wound treatment

All the animal experimental protocols were reviewed and approved by the Institutional Animal Care and Use Committee at Nanjing Medical University (IACUC-2604095). In vivo fluorescence imaging showed that, after 808-nm laser irradiation, BBT-Py^+^ NPs@SchB@CSH achieved active enrichment at the diabetic bacterial wound site. The fluorescence signal at the wound site peaked 2 h post-administration and essentially disappeared by 12 h in the laser-treated group (Fig. 7A). Further observation up to 48 h revealed minimal distribution of BBT-Py^+^ NPs in major metabolic organs, such as the heart, liver, spleen, lungs, and kidneys, suggesting gradual degradation and metabolism after targeted wound enrichment (Fig. 7B). The degradation behavior of this nanosystem likely exhibits microenvironment-responsive characteristics: Infection-induced inflammation generates ROS, which can degrade the outer gel and promote sustained Sch B release, ultimately leading to their clearance from the body. In the short-term treatment study (Fig. 7C), compared to the control group, the Sch B@SBMA alone group, and the group without laser treatment, the BBT-Py^+^ NPs@SchB@CSH + Laser group exhibited thinner and drier scabs on the wound by day 2 (Fig. 7D). Hematoxylin and eosin (H&E) staining showed a significant increase in inflammatory cell infiltration, indicating that this material, when combined with laser treatment, can transiently promote inflammation in the short term, thereby potentially accelerating the wound healing process (Fig. 7E and Fig. S16A). Immunofluorescence results further confirmed this conclusion, with higher CD86 expression observed (Fig. 7F and Fig. S16B).

**Fig. 7.**
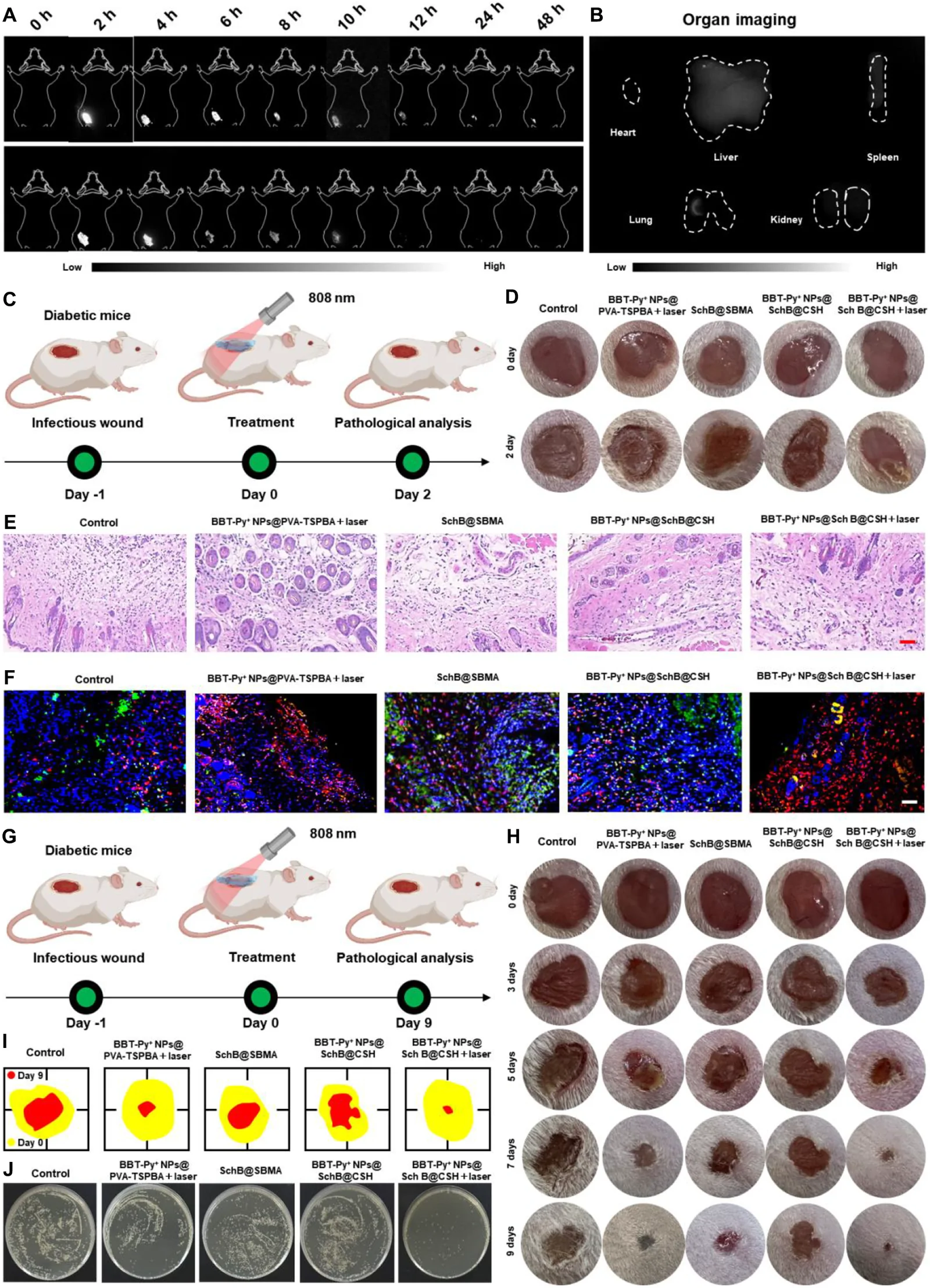
(A) NIR-II fluorescence imaging of diabetic bacterial-infected wounds at different time points (0 to 48 h), showing enrichment and distribution of BBT-Py^+^ NPs@SchB@CSH and BBT-Py^+^ NPs@SchB@CSH + HP at the wound site. (B) Fluorescence imaging of isolated major organs (heart, liver, spleen, lung, kidney) to evaluate the in vivo distribution and metabolism of BBT-Py^+^ NPs. (C) Schematic diagram of the short-term treatment experiment for diabetic bacterial-infected wounds. (D) Wound healing photos of different treatment groups on days 0 and 2. (E) H&E staining of pathological sections of the wound tissue (day 2). (F) Immunofluorescence staining (day 2) for CD86 (M1 macrophages, red) and CD206 (M2 macrophages, green) to evaluate macrophage polarization and anti-inflammatory repair capacity in each group. (G) Schematic diagram of the long-term treatment experiment for diabetic bacterial-infected wounds. (H) Wound healing images of different treatment groups on days 0, 3, 5, 7, and 9. (I) Overlay images of the remaining wound areas of each treatment group. (J) Bacterial colony count of wound tissue homogenate (day 9), quantitatively evaluating the antibacterial effect of each group. Irradiation was performed at 808-nm wavelength, 1.0 W cm^−2^ power density, and for 10 min.

In the long-term treatment study (Fig. 7G), the BBT-Py^+^ NPs@SchB@CSH + Laser group exhibited a significantly faster wound-healing rate than the other groups, with the wound nearly completely closed by day 9 (Fig. 7H). ImageJ analysis of wound area sizes across different treatment groups further highlighted the excellent wound-healing effect of BBT-Py^+^ NPs@SchB@CSH + Laser (Fig. 7I). Plate colony counts verified its excellent antibacterial effect (Fig. 7J and Fig. S17). These results indicate that this nanosystem effectively overcomes the core challenges of persistent infection, excessive inflammation, and insufficient vascular regeneration in diabetic bacterial wound healing through photothermal–chemical synergy and multi-dimensional wound healing promotion, offering a potential new strategy for clinical treatment.

Based on the wound-healing process, the core–shell gel system constructed in this study is expected to significantly promote the repair of infected wounds in diabetic mice through the synergistic effects of NIR light-mediated efficient antibacterial activity, immune regulation, and remodeling promotion (Fig. 8A). Histological and molecular analyses further supported these findings. H&E staining (Fig. 8B and Fig. S18) showed more complete epithelial regeneration, more regular granulation tissue arrangement, and significantly reduced inflammatory cell infiltration in the BBT-Py^+^ NPs@SchB@CSH + Laser group. Masson staining (Fig. 8B) confirmed more abundant and orderly collagen deposition in this group, indicating superior tissue remodeling and repair capacity compared to other treatment groups. Immunohistochemistry (Fig. 8C to E) revealed a significantly lower proportion of TNF-α and IL-6-positive cells in the BBT-Py^+^ NPs@SchB@CSH + Laser group, indicating effective inhibition of pro-inflammatory factor expression and alleviation of the inflammatory response in the wound area. Immunofluorescence staining and quantitative analysis (Fig. 8F and G) showed a significantly higher CD206/CD86-positive cell ratio in this group, indicating a more pronounced polarization of macrophages toward the anti-inflammatory, pro-reparative M2 subtype, thereby promoting tissue repair. Furthermore, CD31 immunohistochemistry (Fig. 8H and Fig. S19) demonstrated enhanced expression of vascular endothelial markers in this group, suggesting improved angiogenic capacity and a favorable microenvironment for wound healing. In summary, BBT-Py^+^ NPs@SchB@CSH, upon laser trigger, accelerate the healing of bacterial-infected wounds by inhibiting inflammation, regulating macrophage polarization, and promoting collagen deposition and angiogenesis.

**Fig. 8.**
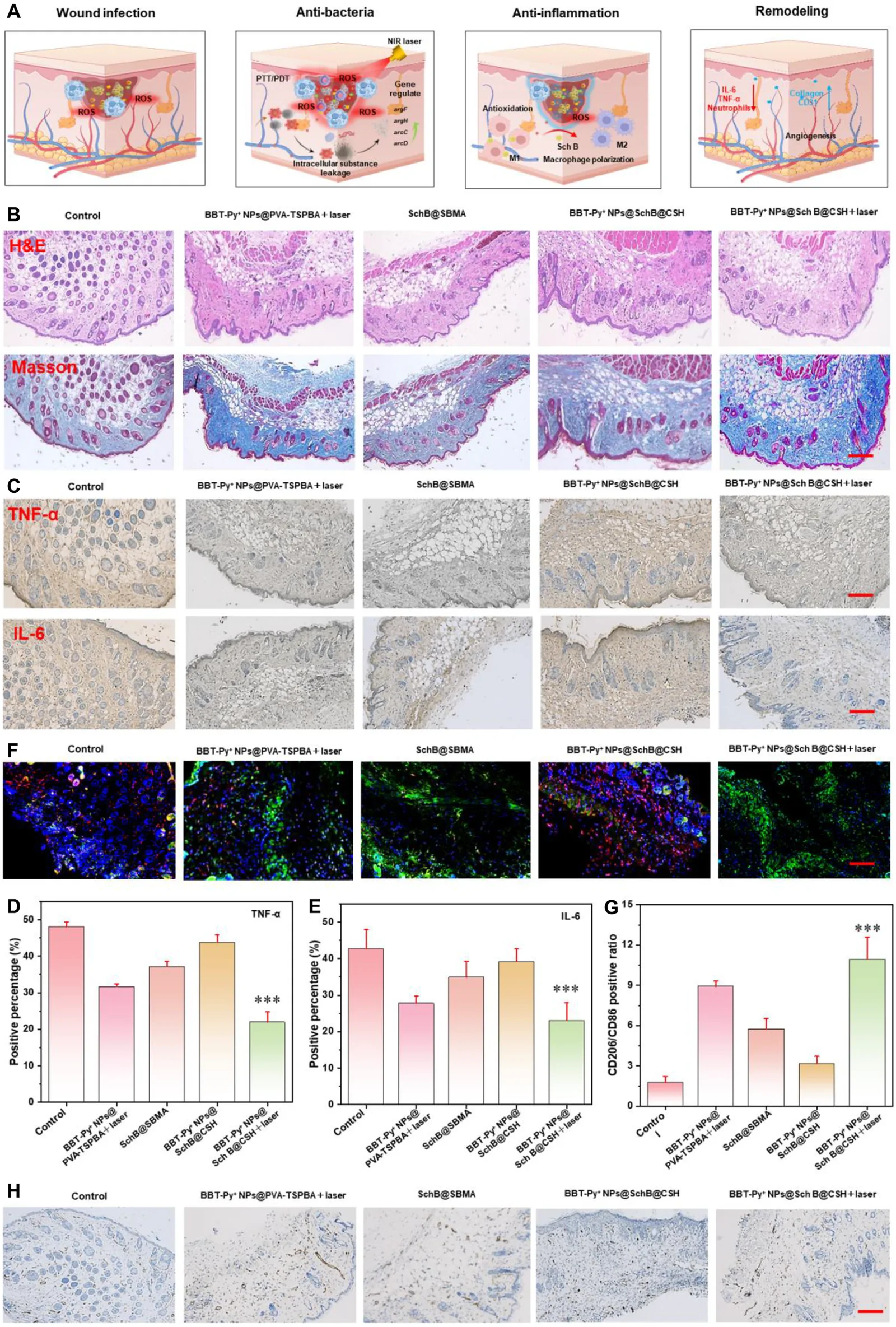
(A) Schematic diagram of wound repair in diabetic mice. (B) H&E and Masson-stained sections of wound tissues from different treatment groups (scale bar, 100 μm). (C) Immunohistochemical staining of TNF-α and IL-6 at the wound sites of each group on day 9. Quantitative analysis of the immunohistochemical staining showing the proportion of (D) TNF-α and (E) IL-6, evaluating the intensity of the inflammatory response. (F) Immunofluorescence staining (CD86, red; CD206, green; 4′,6-diamidino-2-phenylindole, blue), showing the distribution and polarization state of M1/M2 type macrophages in the wound tissues. (G) Quantitative analysis of the ratio of CD206/CD86-positive cells, reflecting the degree of macrophage polarization toward M2 in each group. (H) Immunohistochemical staining for CD31 in the wound tissues of each group. Irradiation was performed at 808-nm wavelength, 1.0 W cm^−2^ power density, and for 10 min. Error bars represent mean ± SD. ***P < 0.001.

To evaluate the in vivo biocompatibility of BBT-Py^+^ NPs@SchB@CSH, we conducted a comprehensive analysis on healthy mice, including hematological and biochemical assays, as well as histopathological examination of key organs. Blood samples were collected from mice 7 d after intravenous injection of phosphate-buffered saline or BBT-Py^+^ NPs@SchB@CSH. We analyzed key liver markers, such as alanine aminotransferase (ALT) and aspartate aminotransferase (AST), as well as kidney markers, including creatinine, uric acid, and blood urea nitrogen. The results showed negligible differences in liver and kidney function between the BBT-Py^+^ NPs@SchB@CSH and control groups 7 d post-injection (Fig. S20), indicating good blood compatibility and no significant functional impairment in mice. H&E staining of major organs, including the heart, liver, spleen, lungs, and kidneys, showed no signs of systemic toxicity 7 d post-injection (Fig. S21).

## Conclusion

In this study, we developed a dual-layer zwitterionic hydrogel system with spatiotemporally controlled therapeutic functions, providing an innovative integrated platform for the precise treatment of bacterial-infected wounds. The outer hydrogel, loaded with BBT-Py^+^ NPs, confers synergistic photothermal–photodynamic antibacterial activity. The inner hydrogel, encapsulating the natural antioxidant Sch B, enables the system to scavenge excess ROS, alleviate oxidative stress, and modulate the immune microenvironment. An ROS-sensitive crosslinking interface connects the 2 layers. Upon laser irradiation, ROS generated by the outer layer via photodynamic action degrade this interface, triggering the controlled release of Sch B from the inner layer and achieving spatiotemporally ordered treatment: antibacterial debridement followed by anti-inflammatory repair. In vivo experiments confirmed that, following laser irradiation, this spatiotemporal strategy controlled infection via the outer layer’s antibacterial effect and promoted epithelial regeneration and collagen deposition via the inner layer’s anti-inflammatory effect, thereby synergistically inducing angiogenesis. This approach successfully addressed the core challenges of persistent infection, excessive inflammation, and insufficient vascular regeneration in diabetic bacterial-infected wound healing. Therefore, the dual-layer multimodal hydrogel system developed in this study offers a novel and efficient solution for the precise, staged treatment of bacterial-infected wounds, demonstrating promising potential for clinical translation.

## Data Availability

Data are available on request from the authors.
