# Supplementary material for: Spatiotemporally Regulated Pro-/Anti-Inflammatory Transition via Reactive Oxygen Species-Sensitive Core–Shell Hydrogel for Diabetic Infected Wound Healing
Source: Biomater Res. 2026 Oct 15;30:0432. doi: 10.34133/bmr.0432 (PMC13669506; doi:10.34133/bmr.0432)
Supplement: Supplementary file 1 [file bmr.0432.f1.docx]

*Supporting information*

Spatiotemporally Regulated Pro‑/Anti‑Inflammatory Transition via Reactive Oxygen Species‑Sensitive Core‑Shell Hydrogel for Diabetic Infected Wound Healing

**Materials and Methods**

**Photothermal properties**

A series of BBT-Py^+^ NPs@SchB@CSH solutions at concentrations of 0, 50, 100, 150, and 200 μg/mL were prepared using deionized water as the solvent. 1.0 mL of each solution was transferred into a 1.5 mL centrifuge tube, and the bottom of the tube was irradiated with an 808 nm near-infrared laser. The temperature changes of the solutions within 10 min were recorded using a FLIR ONE Pro thermal imager. Additionally, the temperature changes under different laser power densities (0.2, 0.4, 0.6, 0.8, and 1.0 W/cm^2^) were investigated for 10 min. To assess photothermal stability, the 200 μg/mL BBT-Py^+^ NPs@SchB@CSH solution was irradiated at 1.0 W/cm^2^ for 10 min, after which the laser was turned off. After the solution cooled naturally, the heating-cooling cycle was repeated four additional times (a total of five cycles) to evaluate photothermal stability. The photothermal conversion efficiency (*η*) was calculated using the first cooling curve according to the following equation:

$$\eta=\frac{\mathrm{hA}\left( T_{\max}-T_{\mathrm{sur}} \right)-Q_{s}}{I(1-{10}^{-A_{808}})}$$

where h is the heat transfer coefficient, A is the surface area of the sample solution, T_max_ and T_sur_ are the maximum temperature achieved after 10 min of irradiation at 1.0 W/cm^2^ and the ambient temperature, respectively, Qs is the heat absorbed by the solvent, I is the laser power corresponding to the highest temperature (1.0 W/cm^2^), and A_808_ is the absorbance of the 200 μg/mL BBT-Py^+^ NPs@SchB@CSH solution at 808 nm. The value of hA was obtained from the following relationship:

$$hA=\frac{m_{s}\cdot c_{s}}{\tau_{s}}$$

In the formula, m_s_ and c_s_ refer to the mass of the BBT-Py^+^ NPs@SchB@CSH solution used in the test and the specific heat capacity of the solvent (4.2 J·g^-1^·k^-1^); τ_s_ is calculated using the following formula:

$$t=-\tau_{s}\ln\frac{T-T_{\mathrm{sur}}}{T_{\max}-T_{\mathrm{sur}}}=-\tau_{s}ln\theta$$

Plotting time t against -lnθ, τs represents the slope of the fitted line, and by calculating further, the photothermal conversion efficiency can be obtained.

**Generation and scavenging of ROS**

For ROS generation detection, 2,3-dihydrodiphenyl-1,10-phenanthroline (DPBF, 10 mM) was used as a probe. The solution was irradiated with an 808 nm laser (1.0 W/cm^2^) for 0-10 min, and the absorbance at 410 nm was recorded. Similarly, 3,3',5,5'-tetramethylbenzidine (TMB, 10 mM) was used as a probe under the same irradiation conditions, and the absorbance at 652 nm was recorded. To evaluate the antioxidant activity of Sch B, different concentrations of Sch B (10-50 μg/mL) were tested using commercial kits: H_2_O_2_ kit (ammonium molybdate method, 405 nm), ·OH (TMB-Fenton method, 652 nm), ·O_2_^-^ (WST-1 method, 450 nm), ABTS (405 nm), and GSH (DTNB method, 412 nm).

RAW 264.7 cells were pretreated with LPS for 4 h and then incubated with different materials for 24 h. Subsequently, the cells were stained with 10 μM DCFH-DA in the dark for 30 min, then washed three times with PBS. Fluorescence intensity was observed using a confocal microscope (Zeiss LSM 880) with excitation/emission wavelengths of 488/525 nm. Moreover, flow cytometry (BD FACSCanto II) was used to quantitatively measure ROS. In brief, first, cell debris and abnormal events were excluded through FSC-A/SSC-A; then, single-cell populations were selected based on the FSC-A/FSC-H relationship; finally, intracellular ROS analysis was performed on the DCFH-DA positive cell population.

**Evaluation of antibacterial performance *in vitro***

*S. aureus* (ATCC 25923) was cultured in LB medium at 37 ℃ in an orbital shaker until the logarithmic growth phase. The bacterial suspension (1 × 10^7^ CFU/mL) was incubated with different materials for 6 h. For groups requiring laser irradiation, the samples were exposed to an 808 nm laser (1.0 W/cm^2^, 10 min) under the corresponding conditions. After treatment, the suspension was serially diluted, spread on LB agar plates, and incubated at 37 ℃ for 12 h to count colonies.

Live/dead staining: The bacteria were stained with PI (100 μg/mL) in the dark for 30 min, washed with PBS three times, fixed with 2.5% glutaraldehyde for 2 hours, and observed under a fluorescence microscope. The bacterial suspension (1 × 10^8^ CFU/mL) was co-cultured with the materials in a 24-well plate at 37 °C for 7 days (the medium was replaced every 24 hours). On the 7th day, the laser irradiation group was exposed to an 808 nm laser (1.0 W/cm^2^, 10 min), and DAPI/PI staining was performed in the dark for 30 min, after which the samples were observed under a confocal microscope.

After crystal violet staining, the stained biofilms were dissolved in 33% acetic acid, and the optical density at 590 nm was measured. The bacterial suspensions of the control group and the BBT-Py^+^ NPs@SchB@CSH+HP treatment group were centrifuged (4000 rpm, 5 min), washed with PBS, rapidly frozen in liquid nitrogen for 15 min, and stored at -80 °C for testing (Genepioneer Biotechnologies).

**Cytotoxicity**

RAW 264.7 cells (1 × 10^4^ cells/well) were seeded in 96-well plates and allowed to adhere for 24 h. The cells were then incubated with different materials for 24 h. Cell viability was assessed using the CCK-8 assay (Jiangsu Keygen Biotech Co., Ltd.) by measuring the absorbance at 450 nm. For live/dead staining, cells were incubated in the dark for 30 min with calcein-AM (2 μM) and PI (8 μM), then observed under a fluorescence microscope.

**Macrophage polarization**

RAW 264.7 cells (1 × 10^6^ cells/well) were seeded in 6-well plates. After 24 h of adhesion, they were treated with different materials for another 24 h. The cells were then collected and stained with CD86-FITC and CD206-PE antibodies at 4 ℃ in the dark for 1 h. Flow cytometry (BD FACSCanto II) was performed, and the data were analyzed using FlowJo software to determine the positive rates and average fluorescence intensities. The representative flow cytometry gating strategy: debris and cell aggregates were excluded according to the FSC‑A vs SSC‑A dot plot, and single cells were further gated by FSC‑H vs FSC‑A. Subsequently, the target macrophage populations were gated for the analysis of CD86‑FITC (M1 marker) and CD206‑PE (M2 marker) signals.

Total RNA was extracted using the TRIzol reagent (Jiangsu Keygen Biotech Co., Ltd), reverse transcribed using the PrimeScript RT Reagent Kit, and qPCR was performed using SYBR Green Master Mix on a QuantStudio 5 system. GAPDH was used as the internal reference, and the relative expression levels of *TNF-α*, *IL-6*, *iNOS*, *Arg-1*, etc. were calculated using the 2^-^ΔΔCt method.

Cells from the PBS control group and the BBT-Py^+^ NPs@SchB@CSH treatment group (1 × 10^6^ cells/well) were centrifuged, rapidly frozen in liquid nitrogen, stored at -80 ℃, and sent for testing.

**Antimicrobial and wound healing effects *in vivo***

Male BALB/c mice (6–8 weeks old) were fasted for 4 consecutive days and then intraperitoneally injected with streptozotocin (60 mg/kg/day). One week later, mice with blood glucose levels >16.7 mM were considered diabetic and used in subsequent experiments. After successful modeling, a full-thickness skin defect (1.5 cm diameter) was created on the back of each mouse, and 200 μL of *S. aureus* suspension (1 × 10^8^ CFU/mL) was inoculated into the wound. After 24 h, the animals were numbered and randomly assigned to 5 treatment groups (n = 5): (1) Control, (2) BBT-Py^+^ NPs@PVA-TSPBA + Laser, (3) Sch B@SBMA, (4) BBT-Py^+^ NPs@SchB@CSH, and (5) BBT-Py^+^ NPs@SchB@CSH + Laser. For the laser groups, the wounds were exposed to 808 nm irradiation (1.0 W/cm^2^, 10 min). Wound healing was monitored by taking photographs on days 0, 1, 3, 5, 7, and 9, and the wound images were collected and anonymously coded. Researchers unaware of the experimental grouping used ImageJ software to measure wound area and calculate the wound closure rate.

Mice were sacrificed on day 9, and wound tissues were collected. Tissue homogenates were diluted and plated on LB agar for colony counting. Additionally, wound tissues were fixed, embedded in paraffin, sectioned, and stained with H&E, Masson's trichrome, and immunofluorescence stains, and observed under an optical microscope. For *in vivo* imaging, mice with wounds treated with BBT-Py^+^ NPs@SchB@CSH (with or without laser) were imaged using an IVIS Spectrum imaging system at 0, 2, 4, 6, 8, 10, 12, 24, and 48 h after treatment.

**Supplementary Figures**

**Figure S1.** Synthesis and ^1^H-NMR of BBT-Py.

**Figure S2.** Synthesis of BBT-Py^+^-Me_2_.

**Figure S3.** (A) Zeta potential diagram of BBT-Py^+^ NPs. (B) Particle size plots in PBS and DMEM at different time points.

**Figure S4.** Changes in storage modulus (G') and loss modulus (G'') of PVA-TSPBA hydrogel over time.

**Figure S5.** Relative change in ultraviolet absorption intensity of DPBF at 410 nm under conditions of BBT-Py^+^ NPs@SchB@CSH.

**Figure S6.** Infrared thermograms of different concentrations of BBT-Py^+^ NPs under 808 nm laser irradiation at a power density of 1.0 W/cm^2^. (B) Infrared thermograms of BBT-Py^+^ NPs under 808 nm laser irradiation at different power densities.

**Figure S7.** Infrared thermograms of PVA-TSPBA@Sch B@CSH, BBT-Py^+^ NPs, and BBT-Py^+^ NPs@SchB@CSH under 808 nm laser irradiation at a power density of 1.0 W/cm^2^.

**Figure S8.** Stability of BBT-Py^+^ NPs@SchB@CSH under repeated irradiation cycles.

**Figure S9.** (A) Corresponding reciprocal plot. Steady-state constants of Sch B@SBMA CAT-like activity. (B) Steady-state constants of Sch B@SBMA CAT-like activity.

**Figure S10.** (A) Free radical scavenging efficiency of BBT-Py^+^ NPs@SchB@CSH before and after laser irradiation. (B) ·OH, (C) H_2_O_2_, and (D) ·O_2_^-^ clearance ability of BBT-Py^+^ NPs@SchB@CSH before and after laser irradiation. Scale bar = 50 μm. Data are presented as mean ± SD (n = 3).

**Figure S11.** (A) Cell survival ability of RAW264.7 cells under different treatments. (B) Live/dead cell staining images of RAW264.7 cells after different treatments. Data are presented as mean ± SD (n = 3).

**Figure S12.** **Results of quantitative analysis of fluorescence intensity in different treatment groups.** Data are presented as mean ± SD (n = 3). ****p* < 0.001.

**Figure S13.** Residual amount of *S. aureus* biofilm after 24 hours of different treatments. Data are presented as mean ± SD (n = 3). **p* < 0.05 and ****p* < 0.001.

**Figure S14.** Dry weight of bacterial biomass after 7 days of different treatments. Data are presented as mean ± SD (n = 3). ****p* < 0.001.

**Figure S15.** qPCR analysis of expression levels of genes related to inhibition of bacterial biofilm formation, *sspB*, *qoxB*, *arcC*, and *arcD* after different treatments. Data are presented as mean ± SD (n = 3). ****p* < 0.001.

**Figure S16.** (A) Quantitative analysis of H&E staining of neutrophil regions in mouse skin after 48 hours. (B) Positive rate of CD206/CD86 in mouse skin tissue after 48 hours. Data are presented as mean ± SD (n = 3). ****p* < 0.001.

**Figure S17.** Survival rate of S. aureus in mouse skin tissue homogenate after 9 days of different treatments. Data are presented as mean ± SD (n = 3). ****p* < 0.001.

**Figure S18.** Quantitative analysis of H&E staining of mouse skin neutrophil regions after 9 days. Data are presented as mean ± SD (n = 3). ****p* < 0.001.

**Figure S19.** Immunohistochemical staining of CD31 in wounds of each group on the 9th day. Data are presented as mean ± SD (n = 3). ****p* < 0.001.

**Figure S20.** Biological safety assessment of BBT-Py^+^ NPs@SchB@CSH 24 hours after intravenous injection. Blood samples were collected 7 days after intravenous administration of PBS or BBT-Py^+^ NPs@SchB@CSH to ICR mice, and liver function markers (ALT, AST, and ALB) and kidney function markers (CREA, UA, and BUN) were used to evaluate their biological safety and blood compatibility. Data are presented as mean ± SD (n = 3).

**Figure S21.** H&E staining of the heart, liver, spleen, lungs, and kidneys of healthy ICR mice after intravenous injection of BBT-Py^+^ NPs@SchB@CSH for 7 days. Scale bar = 100 μm.
